# Opening Up the Politics of Knowledge and Power in Bioscience

**DOI:** 10.1371/journal.pbio.1001233

**Published:** 2012-01-03

**Authors:** Andy Stirling

**Affiliations:** SPRU and STEPS Centre, University of Sussex, Brighton, United Kingdom; The London School of Economics and Political Science, United Kingdom

## Abstract

Public engagement is not in tension with science, but actually a way to be more rigorous - as well as more democratic - about social choice of biotechnology.

Recent years have seen growing worldwide discussions, experiments, and expectations around various kinds of public engagement in the biosciences. This is especially so, in the governance of biotechnology—in research policy, risk regulation, and adoption of new innovations. How one defines public engagement necessarily affects the course of political, media, and civil society debate on these issues. Yet critics and even some proponents often misunderstand underlying rationales and imperatives for engagement [1]. Strong opposition persists on the part of some policymakers in the ostensible name of science, even to the most modest forms of citizen participation in decisions about regulation or research [2]. Where dialogue is supported between scientists, policy makers, stakeholders, and members of the public, it is often for contrasting reasons [3]—reflecting motivations of some leading figures in science governance to control, as much as respect, contending public interests [4]. Prominent experts have questioned whether ordinary people have the right or even the ability to engage on complex technical issues [5]. Attempts to include stakeholders are criticized as slowing down innovation [6]. Some scientists fear that irrational anxieties over particular issues mean that public engagement will lead to indiscriminately technophobic or anti-science results [7]. How might we interpret these attitudes and controversies and better understand why public engagement matters? What are the practical policy consequences?

This paper identifies different grounds for supporting particular elements of public engagement, irrespective of context. It describes how diverse qualities of participatory practice arise in different circumstances. The starting point is that the realities of technological change—particularly as they relate to policy making—demand a move away from traditional exclusive, specialist approaches. This means relinquishing the blanket pro-innovation rhetoric used by many in high-level policy making: portraying technological progress as what Lord Alec Broers (as President of the Royal Academy of Engineering) described in his globally broadcast BBC Reith lectures as a “race to advance technology”—a single track to an essentially inevitable future [8]. This linear notion conceals the continually branching nature of technological change. It hides the ways important political choices over alternative directions for innovation are made at every juncture—and should be as subject as other areas of policy, to democratic participation and accountability. In this sense, then, various kinds of public engagement in the biosciences can be seen to offer means to reconcile tensions between the otherwise-estranged Enlightenment values of science and democracy. In short, greater public engagement offers an opportunity to be more rigorous about the uncertainties in bioscience innovation and more accountable about the exercise of power.

In introducing this series of commentaries [9], the editors follow others in distinguishing between three broad rationales and imperatives for public engagement [10],[11]. First, a substantive approach tries to identify the so-called “best” outcomes—trajectories for technology that respect broadly shared public values like maximizing public benefits, reducing health impacts, increasing environmental sustainability, or enhancing wellbeing. Details are ambiguous and contestible, but such widely debated aspirations do offer nontrivial, generally self-evidently positive ends. Second, by contrast, instrumental objectives presume (often implicitly) that a specific outcome is desirable—one favoured by particular interests and perspectives (for instance, individual businesses, agencies, or pressure groups). These approaches therefore focus simply on the means towards unquestioned ends—like fostering public understanding, trust, reputation or acceptance, or giving voice to opposition—all with respect to some particular option or institution. Whether or not one approves of such closure from ends to means, depends on the inclination to support the particular ends thereby privileged. Finally, normative approaches are not primarily concerned with outcomes at all—neither as ends nor means—but with the participatory processes themselves. These value such qualities as independence, openness, accessibility, legitimacy, and accountability [12]. Normative evaluations of public engagement processes can also depend on whether they are structured or spontaneous, deliberative or expressive, invited or uninvited [13]. Thus, decisions about how best to view, design, or choose among modes of public engagement depend on perspective as well as context.

But in asking “whether, and under what conditions, it is possible to engage the public in scientific issues in meaningful ways?” [9], the editors risk being misunderstood. This might be taken to imply that, whilst the public may display divergent views on particular technologies, this is not the case when it comes to more general perspectives on the role of public engagement concerning these technologies. Although conclusions may vary by case, there is an implication (at least in principle) that objectively correct answers exist at the most general level. Such an impression reflects a current trend under which public engagement has grown increasingly structured and subject to accreditation, institutionalisation, professionalization, and managerial evaluation. Together, this trend tends to suppress the intrinsically political dimensions in the governance of science, which requires flexibility to accommodate diverse values and viewpoints. Yet, in reality, the answers to the questions posed in this series are partly in the eyes of beholders—hinging not only on particular conditions, but also on divergent political perspectives under any given condition [14].

For instance, in deciding which innovations to pursue in agriculture (technological or social), it cannot be assumed that any one aim is paramount—whether the issue is respecting the cultural attributes of food, maximizing world protein production, commercial revenues in supply chains, combating climate change, or sustaining hard-pressed livelihoods. All are valid concerns, but not all can be maximized together. Although participation may improve mutual understanding and appreciation among stakeholders, even the most inclusive or co-operative practices cannot definitively reconcile underlying contrasting interests. Yet such diverging interests have implications not only for which innovations to chose, but also for what counts as “appropriate” participatory practice. These political dimensions of public engagement can easily be missed by all interested parties—proponents with romantic visions of engagement, practitioners with diverse methodological commitments, sponsors with contending expedient interests, and purveyors of blanket criticism of public engagement, who fail to discriminate between the crucial details of process and context [15].

One way to transcend these differences (without presuming to resolve them one way or another), is to begin with the commitment that governance of bioscience should be informed by the most appropriate knowledges. In public engagement, as in other forms of policy appraisal (like risk assessment or cost-benefit analysis), this raises questions over the nature of different knowledge bases. Figure 1 offers a stylized picture of fundamental ways in which knowledge conditions bearing on bioscience policy may be seen as problematic—and points in each case to illustrative roles for engagement. For ease of understanding, Figure 1 is structured according to the conventional parameters of expert risk regulation: probabilities and magnitudes [16]. Each presents a distinct dimension under which anyone can be more or less confident in their knowledge. It is important to appreciate that this is not a taxonomy of conditions under which knowledge is objectively better or worse. The point is rather that: how a given body of knowledge is regarded is inherently subjective. In particular, Figure 1 shows how public engagement is relevant under all conditions and perspectives, although specific features may vary in significant ways.

**Figure 1:**
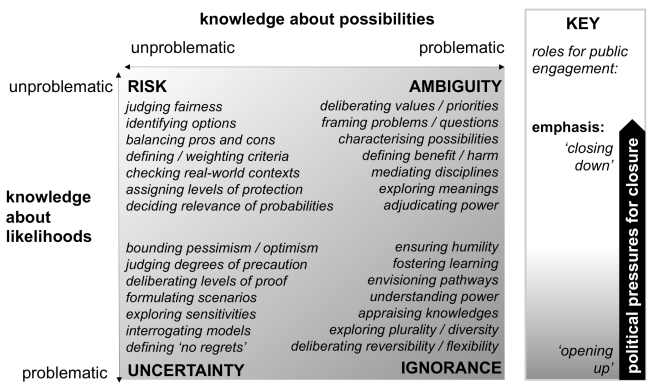
Roles for public engagement under different knowledge conditions.

The top left quadrant shows the classic condition of risk—where there is felt to be complete, high quality knowledge about both magnitudes of possible outcomes and their respective probabilities. Each dimension can be quantified and multiplied to yield formal expressions of risk. This in turn allows optimization across trade-offs between contending objectives, values, and possibilities—thus, the argument goes, informing better policy [17]. One might think that this kind of conventional calculative risk assessment would find little value from public engagement. And it is on this basis that a stark divide is routinely drawn between the ostensibly objective, rational expertise employed in risk assessment and the apparently less informed, more emotional risk perceptions of the public. In this view, public risk perceptions must be managed as spurious departures from the rational norms of experts.

This well-established rigid separation between assessment and management of risk is consistent with the “one track” view that knowledge and innovation in any given area must necessarily follow single optimal trajectories. If one believes that science discovers facts and that facts determine technology, then there is little latitude for meaningful social engagement on the direction of technology change. Under this narrow “risk” view, the function of public engagement is merely instrumental, a means to implement inevitable progress. Engagement is then useful only for securing public understanding, trust, and acceptance of whatever technological developments happen to be currently emerging—that is, for bringing public perceptions in line with risks as defined by the experts.

Even under such constrained instrumental risk-based views, however, bioscience presents a wide range of more substantive governance challenges. These challenges are clearly not purely calculable but involve value judgments. How to: Decide which technology or policy options to include? Weigh contrasting benefits and harms? Balance overall pros and cons? Arbitrate their distribution across society? Determine real-world conditions of use? Derive and aggregate relevant probabilities? Assign optimal levels of protection? Whether acknowledged or not, even the most narrowly science-centric and technocratic notions of risk assessment require qualitative deliberation on such matters. Whether expert or public, subjective judgment is as essential as any kind of objective rationality. This highlights a role for the type of detailed, open, and accountable oversight and validation offered by substantive and normative concepts of public engagement [18].

This case for noninstrumental public engagement further increases under conditions of uncertainty (the lower left of Figure 1). The long-established strict definition of this state of uncertainty is that it affords no firm basis for probabilities [19]. Yet the facility of methods like Bayesian calculus lead to the assertion “…it is always possible to obtain a probability…” [20]. As a result, the term uncertainty is often stretched to describe even relatively tractable conditions under which probabilities yield determinate answers—in effect confusing uncertainty with risk. When practitioners of a particular quantitative method insist on using their favoured techniques even when they are inapplicable, the result is a deep misunderstanding—as prevalent in bioscience as elsewhere—understating indeterminacy and exaggerating the definitive power of calculation [21].

The point is not that it is impossible to assign different subjective probability functions under uncertainty, but that probabilistic reasoning under uncertainty cannot yield a single objectively aggregate value. Here, quantitative methods should not be used to give misleadingly definitive impressions of confidence. Yet they may still offer powerful tools—especially where they acknowledge subjectivity [22]. In other words—under uncertainty—calculation can only serve, not drive, assessments. Scientific rigour demands instead more open-ended forms of uncertainty heuristics, interval analysis, sensitivity testing, and scenario assessment—each requiring attention to the differing conditions that may frame the question at hand [23].

Here the substantive roles for public engagement become immediately clear. It is only through participatory practices catalysed by, focused on, or relevant to, more open-ended methods, that substantive policy appraisal (focusing on salient public concerns) is most likely to be effective. For instance, deliberation can then be more easily directed at key questions such as scrutinizing need, resolving new options, maximizing best case opportunities, ameliorating worst case possibilities, highlighting “no regrets” strategies, or identifying some intermediate precautionary balance. Regulatory experience repeatedly reveals how artificial reduction of uncertainty to risk can compromise public safety when an unforeseen hazard arises. This was the case, for instance, with stratospheric ozone depletion, transmissible spongiform encephalopathies, and endocrine-disrupting chemicals. In each case, early warnings were noticed first outside formal risk assessment. The way to remedy this, is to “broaden out” regulatory appraisal—extending attention to a wider range of options, issues, conditions, uncertainties, scenarios, methods, disciplines, and perspectives than are conventionally included in technical risk assessment. This helps mitigate the obscuring of emerging understandings and early warnings that can be caused in simple reductions to probabilities and magnitudes and aggregating across different circumstances and dimensions [24].

It can be difficult for those wed to probabilitistic approaches, to accept the distinction between risk and uncertainty. So the horizontal axis in Figure 1—highlighting intractabilities in defining possibilities themselves—may be even more unpalatable. These challenges of ambiguity differ from uncertainty, because they apply even after outcomes have already occurred. For example, much of the controversy over genetically modified organisms concerns not the likelihood of some agreed form of harm, but fundamentally different understandings of what harm actually means (e.g., in terms of threats variously to human health, ecological integrity, agronomic diversity, indigenous food cultures, sustainable rural livelihoods, vulnerability to climate change, control of intellectual property, or global industrial distribution). Likewise in other areas, contrasting pictures arise in focusing on different harmful mechanisms, toxic endpoints, or pathogenic vectors [24]. How then does one define, bound, partition, and prioritize different possibilities? What sorts of questions should regulators ask: Do we need this? What would be best? What would be better? What would be safest? What would be safe enough? What would be tolerable? Or (as is routine), is some particular market development, merely “not worse than current worst practice”? Each can yield radically different answers [25].

Although assessment of some specialized questions—involving, for example, incidence of specific occupational disorders, childhood illness, congenital morbidity, environmental disease—may be seen quite fairly as largely a matter for expertise, deciding between and within such questions still requires intrinsically subjective judgments. What kind of expertise can plausibly settle the relative importance of compared levels of, say: Injury or illness? Harm to adults or children? Worker or citizen? Present or future generations? Humans or animals? Here, Nobel-winning work in rational choice theory shows, as a matter of logic, that there exists no general form of analysis that can guarantee uniquely optimal answers across specific cases [26]. The same holds for other kinds of ambiguity: it is misleading to claim that single definitive science-based decisions are possible—science alone cannot reconcile the range of contrasting, plausibly preferable outcomes. Again, substantive public engagement—symmetrically addressing diverse portfolios of choices [27]—offers a path to facilitating both validity and legitimacy under these conditions [28].

Here, substantive public engagement requires not just interactions between different groups of experts but rich varieties of encounter between experts and nonspecialists. Indeed, the terms under which different disciplines themselves engage is itself often at issue. Institutional dominance, conflict, and exclusion all feature prominently in past histories of regulatory failure [24]. When and how, for example, should social scientists play a role? Merely in the final communication of results? In helping elicit options, weights or priorities as inputs? In investigating issues of use, practice, or compliance? Or in illuminating the social processes of science itself, examining the dynamics of knowledge and power? Each holds divergent implications. All are combined in enabling participatory deliberation. Though technical details may be inaccessible to the general public, underlying political dynamics between academic, governmental, and commercial institutions are broadly familiar to nonspecialists, but often marginalised by specialists. In this way, the life sciences are little different to other specialisms such as security, economy, or law—and should arguably be equally subject to democratic accountability. Under ambiguity, public engagement offers a way to integrate the wide diversity of public viewpoints into policymaking with a fine-grain detail that is typically not achievable in parliamentary or legislative proceedings (let alone expert assessment alone).

Beyond risk, uncertainty, and ambiguity lies the final aspect of problematic knowledge in bioscience governance—ignorance (lower right of Figure 1) [29]. This is where public engagement is particularly valuable. Here, the challenge is not just about the prospect of radical surprise—“unknown unknowns” like newly recognized kinds of adverse outcome or harmful mechanism mentioned above [24]. The predicament is further amplified by the way scientific and technological developments—including the fabric of knowledge itself—can be conditioned by expectations and power [11]. This is because research and innovation (social as much as technological) proceed through continually branching choices. Many different disciplines have shown how, once chosen, each pathway becomes channeled in ways that are difficult to reverse. Alternative paths are crowded out, other opportunities foreclosed. History is littered with examples—like QWERTY keyboards, VHS videos, narrow-gauge rail, urban automobiles, AC electricity, light-water reactors, and PC software. Even the most competitive markets repeatedly lock in to retrospectively clearly inadvisable choices. Whilst real world complexities ensure some degree of diversity, the repeated lesson of history is that society cannot commit to any single trajectory without diminishing the potential for others [30]. Artificially blinkered ignorance is itself one of the key mechanisms of closure [21]. And those pathways of change favoured by the least powerful are typically the most excluded [31].

In other words, scientific and technological progress is not about one-track competitive races to discover in each field what is self-evidently better. It is an exploratory process that closes down, as well as opens up, alternative possibilities. And, in a globalising world, the stakes are further raised by corporate concentration and pressures for harmonization and standardization (as championed by the World Trade Organization). For instance, though alternative trajectories are biologically feasible in agricultural seed production—and potentially economically viable and socially realizable—incentive structures for large corporations in global markets favour strategies that assert intellectual property (IP) or otherwise maximize profits in a supply chain. This helps explain the conventional industrial emphasis on hybrid varieties and preference for IP-intensive transgenics. Other technical approaches may also be relatively neglected for narrow commercial reasons, like forms of cisgenics (using similar techniques within species and varieties) or apomixis (allowing greater farmer selection using asexual reproduction) or marker-assisted methods (augmenting conventional breeding with advanced genetics). Equally knowledge-intensive social and institutional innovations are even more disadvantaged—especially those emphasising the interests of marginal groups (like participatory breeding, noncommercial extension practices, or microfinanced indigenous production). In these ways, momentum along particular innovation pathways is driven more by political economy than scientific inevitability. These path-dependent choices are not just about “sound science” and technical optimization, but the exercise of political power [29].

The key to substantive understanding of public engagement as a response to ignorance, then, lies in appreciating these real world dynamics of science and technology choice. Diverse, open, self-defining forms of public engagement offer means equally towards rigorous appreciation and democratic accountability in the social appraisal of innovation pathways. In such ways, the central focus of bioscience governance can expand beyond questions merely about “how much?,” “how fast?,” “how costly?,” or “who leads?”—towards broader, more open-ended, and demanding political challenges around “which way?,” “who says?,” and “why?” This extends far beyond instrumental views of top-down participation supporting regulatory assessment in any given area to optimise a presumed one-track race to the future. Yet this is where much practice and advocacy of public engagement also falls short. By emphasising consensus or settled verdicts, structured, “invited” engagement can thwart genuine substantive public participation as much as any narrow risk assessment—and can also reinforce the closure of discussion around single trajectories. This is especially so, when engagement presumes to settle within highly designed deliberative procedures essentially political matters of choice [11].

A very different picture emerges when public engagement is undertaken not to force consensual prescriptive recommendations, but to map out alternative pathways—revealing the detailed political implications of each [27]. The aim here is to catalyse and provoke—rather than substitute or suppress—wider public discourse. Only in this way, may we hope to reconcile the otherwise contending imperatives of normative legitimacy, substantive rigour, and instrumental efficiency with which this paper began. Decisions will still be made, but they will be more explicitly (and honestly) political: not hiding behind (and subverting) science through simplistic, misleading one-track, “sound science” or “pro-technology” language. So, far from being more protracted or expensive, avoiding pretence at definitive closure can—both in analysis and deliberation—better inform, clarify and streamline political decision making. It is by opening up social choices, that public engagement in policy appraisal can simultaneously enhance robustness (by acknowledging uncertainty, ambiguity, and ignorance) and provide for more transparent accountability (by highlighting judgments).

Despite the many different forms, roles, and perspectives around public engagement, then, it is clear that (in bioscience governance, as elsewhere), the real value of more inclusive participation lies in opening up—rather than closing down—a healthy, mature, accountable democratic politics of technology choice [32]. So, the challenge lies not so much in procedural design, as in the creation of a dynamic new political arena—in which reasoned scepticism is as valued in public debates about technology as it is in science itself. In this way, we may hope to renew and recombine two strangely sundered aspects of the Enlightenment: science and democracy. Far from presenting obstacles (as often implied), it is the emergence of a diverse vibrant new “fifth estate” of practices and institutions around public engagement that best embodies a true Enlightenment vision of progress. Indeed, in bioscience as elsewhere, this exercise of greater social agency over the directions for knowledge and innovation moves beyond enlightenment over the mere possibility of social advance, towards real enablement of a greater diversity of directions for human progress [33].
